# Self-Assembly of a Two-Dimensional Coordination Polymer Based on Silver and Lanthanide Tetrakis-Acylpyrazolonates: An Efficient New Strategy for Suppressing Ligand-to-Metal Charge Transfer Quenching of Europium Luminescence

**DOI:** 10.3390/polym15040867

**Published:** 2023-02-09

**Authors:** Yury A. Belousov, Mikhail T. Metlin, Darya A. Metlina, Mikhail A. Kiskin, Ilya A. Yakushev, Trofim A. Polikovskiy, Ilya V. Taydakov, Andrei A. Drozdov, Fabio Marchetti, Claudio Pettinari

**Affiliations:** 1 Chemistry Department, M.V. Lomonosov Moscow State University, Leninskie Gory Str, Building 1/3, 119991 Moscow, Russia; 2 P. N. Lebedev Physical Institute of Russian Academy of Sciences, Leninsky Prospect 53, 119991 Moscow, Russia; 3 Kurnakov Institute of General and Inorganic Chemistry of the Russian Academy of Sciences, Leninsky Prospekt 31, 119991 Moscow, Russia; 4 School of Science and Tecnology, Chemistry Interdisciplinary Project (ChIP), University of Camerino, Via Madonna delle Carceri, 62032 Camerino, Italy; 5 School of Pharmacy, Chemistry Interdisciplinary Project (ChIP), University of Camerino, Via Madonna delle Carceri, 62032 Camerino, Italy

**Keywords:** lanthanides, luminescence, LMCT quenching, silver, pyrazolonates, europium, heterometallic complexes, coordination polymers

## Abstract

A new strategy for the easy polymerization of anionic [Ln(Q^cy^)_4_]^−^ (HQ^cy^-4-(cyclohexanecarbonyl)-5-methyl-2-phenyl-2,4-dihydro-3H-pyrazol-3-one) into two-dimensional layers of [AgLn(Q^cy^)_4_]_n_ (Ln = Sm, Eu, Gd, Tb and Dy) is proposed by binding the single molecular anions [Ln(Q^cy^)_4_]^−^ to silver cations through the coordination of the pyridinic nitrogen atoms of the pyrazolonate rings. The luminescent properties of [AgLn(Q^cy^)_4_]_n_ have been studied in detail, and it was shown that the previously described low photoluminescence quantum yield (PLQY) of [Eu(Q^cy^)_4_]^−^ is due to Ligand-To-Metal Charge Transfer (LMCT) quenching, which is effectively suppressed in the heterometallic [AgEu(Q^cy^)_4_]_n_ polymer. Sensibilization coefficients for H_3_O[Eu(Q^cy^)_4_], [AgEu(Q^cy^)_4_]_n_, and H_3_O[Sm(Q^cy^)_4_] complexes (n ≈ 1) were estimated via theoretical analysis (also by using Judd-Ofelt theory for Sm^3+^) and PLQY measurements.

## 1. Introduction

Coordination polymers (CPs) of various dimensions, including porous MOF materials, are attracting unrelenting interest from materials scientists and chemists due to the combination of unique functional properties (magnetic, catalytic, or luminescent) of individual molecular units with the stability of polymeric materials and the presence of pores and channels capable of reversible sorption of guest molecules [1,2]. Undoubtedly, the respectable place in the chemistry of coordination polymers is occupied by lanthanides derivatives [3,4,5]. The directed synthesis of CPs based on the Ln^3+^ ions is complicated by high and variable values of coordination numbers, as well as the absence of preferred polyhedra for *4f*-elements [6,7]. At the same time, the luminescent properties of lanthanide complexes, due to the unique features of the electronic configuration, find application in the creation of various materials for light-emitting devices (LEDs) [8,9,10,11], biological luminescent labels [12,13,14], in thermometry [15,16,17] and in chemical sensors [18,19,20,21,22,23].

The most common approach to solve all these problems involves the use of antenna sensitization of lanthanide luminescence, i.e., inclusion in the complex of organic ligands capable of effectively absorbing exciting radiation and transmitting it to the emission center, the lanthanide cation [6,24,25,26]. Among the anionic ligands, the most effective luminescence sensitizers, β-diketones stand out, which, nevertheless, usually form mononuclear complexes [Ln(β-diketone)_3_(L)_1–2_] or molecular anions [Ln(β-diketone)_4_]^−^, which are soluble in typical organic solvents and have moderate chemical stability [27]. The monomeric nature of most studied Ln^3+^ diketonates can complicate functional applications in a number of problems, for example, in the creation of sensor materials [22,23]. To create oligomeric and polymeric lanthanide coordination compounds, it is necessary to use polydentate ligands, such as carbocyclic [28] and heterocyclic [5] aromatic carboxylic acids, which often have absorption maxima with λ < 300 nm and have low absorption in the near UV region (300–400 nm). Less than 10% of the approximately 4000 structures of lanthanide diketonates deposited in the Cambridge Crystal Data Centre (CCDC) have a polymeric structure. However, some methods are now known for linking diketonate complexes into coordination polymers. This goal can be achieved by introducing additional donor atoms into the ligand structure (as, for example, in pyrazole- [29], imidazole- [30], or pyridine-containing [31,32,33,34,35] β-diketones), or by using additional linker ligands, such as bis(diphenylphosphine oxides) [36,37,38,39,40,41,42], 4,4’-bipyridyl [43] or its N-oxide [44], quinone-based ligands [45], 2,2’-bipyrimidine [46] etc. Tetrakis anions [Ln(β-diketone)_4_]^−^ can be polymerized by forming alkali metal salts under non-aqueous conditions [47,48,49]. In most of the mentioned cases, the dimensionality of the polymer is limited by the coordination number of the lanthanide: if in a typical complex with CN = 8 three diketonate ligands occupy six positions, then the polymer is forced to be linear.

In the chemistry of lanthanides, among β-diketone-related ligands, a special role is played by acylpyrazolones [50,51,52] containing a heterocyclic pyrazolone fragment as a component of the β-diketonate system. This is due to the high energies of the triplet level in comparison with common aromatic β-diketones, such as dibenzolylmethane and thenoyltrifluoroacetone, which make it possible to effectively sensitize the luminescence of Tb^3+^ [53,54,55,56] and Dy^3+^ [57,58,59] ions. Acylpyrazolonates of these metals have been repeatedly tested as emitting layers in OLED devices [53,54,56,58] showing high efficiency values. In all cases, the coordination of lanthanides occurs at the acylpyrazolonate Q^−^ site with the formation of neutral tris complexes [LnQ_3_(L)_1–2_] [37,57,60,61,62,63,64,65,66] or tetrakis anions [LnQ_4_]^−^ [57,67,68,69], or dimers [LnQ_3_]_2_ [70,71,72].

Previously, coordination polymers based on rare-earth acylpyrazolonates were obtained only through the use of bridged ligands of the phosphine oxide series [36,37]. In the present paper, we report a fundamentally new approach to the creation of coordination polymers due to the binding of [Ln(Q^cy^)_4_]^−^ anions by Ag^+^ cations through nitrogen atoms of pyrazole rings with the formation of 2D-MOF [AgLn(Q^cy^)_4_]_n_, where Ln = Sm-Dy, and HQ^cy^ = 4-(cyclohexanecarbonyl)-5-methyl-2-phenyl-2,4-dihydro-3H-pyrazol-3-one. Such a reaction is possible due to the fact that silver cations, being soft Pearson acids, form complexes with diketonate ligands [73] only with difficulty, preferring coordination with softer donor atoms, for example, the nitrogen atom [73,74]. Moreover, the H_3_O[Ln(Q^cy^)_4_] (Ln = Sm, Tb, Dy) coordination compounds have pronounced ion-centered luminescence with the quantum yields (~2, 56 and 3%, respectively) close to the highest ones to date [75,76]. However, a curiously low value of overall PLQY was obtained for the H_3_O[Eu(Q^cy^)_4_] complex, below the measurement limit. From our investigation we could state that this uncommon result is due to the luminescence quenching via LMCT. Effective suppression of the LMCT processes was accomplished by the new method, provided in our research, of a change-over to polymer complexes with an Ag^+^ ion instead of H_3_O^+^ in the outer coordination sphere. Specifically, we were able to totally prevent the LMCT participation in the luminescent process in the [AgEu(Q^cy^)_4_]_n_ complex with significantly high PLQY increase of up to two orders compared to the H_3_O[Eu(Q^cy^)_4_] one.

## 2. Materials and Methods

### 2.1. Synthesis and Spectroscopic Characterization of the Complexes

The hydroxonium complexes H_3_O[Ln(Q^cy^)_4_] were prepared according to the procedure previously reported [57] for a H_3_O[Dy(Q^cy^)_4_] complex using LnCl_3_ hexahydrates as starting reagents. The isostructurality of the complexes **1–5** was confirmed by powder diffraction (see SI, Figure S1).

**H_3_O[Sm(Q**^cy^**)_4_], 1.** Pale yellow plates, 78%. Anal. Calc. for C_68_H_79_N_8_O_9_Sm: C, 62.69; H, 6.11; N, 8.60; Sm, 11.54%. Found: C, 62.0; H, 6.7; N, 8.5; Sm, 11.9. IR (cm^−1^): 3392 m (ν O–H^H3O+^), 2929 m, 2853 m (ν C-H^Cyclohexyl^), 1662 w (δ O–H^H3O+^), 1612 m (ν C=O^Diketone^), 1593 m, 1584 m, 1499 m (ν C–C), 1400 m, 1235 m, 1145 w, 1078 vs, 1027 s, 902 m, 812 m, 691 w.

**H_3_O[Eu(Q**^cy^**)_4_]**, **2**. Colorless plates, 73%. Anal. Calc. for C_68_H_79_N_8_O_9_Eu: C, 62.62; H, 6.10; N, 8.59; Eu, 11.65%. Found: C, 62.5; H, 6.7; N, 8.6; Eu, 12.0. IR (cm^−1^): 3392 m (ν O–H^H3O+^), 2929 m, 2852 m (ν C-H^Cyclohexyl^), 1662 w (δ O–H^H3O+^), 1614 m (ν C=O^Diketone^), 1593 m, 1584 m, 1499 m (ν C–C), 1400 m, 1232 m, 1145 w, 1078 vs, 1028 s, 902 m, 812 m, 692 w.

**H_3_O[Gd(Q**^cy^**)_4_]**, **3**. Colorless plates, 74%. Anal. Calc. for C_68_H_79_N_8_O_9_Gd: C, 62.36; H, 6.08; N, 8.56; Gd, 12.01%. Found: C, 62.1; H, 6.4; N, 8.4; Gd, 12.2. IR (cm^−1^): 3391 m (ν O–H^H3O+^), 2930 m, 2852 m (ν C-H^Cyclohexyl^), 1661 w (δ O–H^H3O+^), 1616 m (ν C=O^Diketone^), 1592 m, 1584 m, 1500 m (ν C–C), 1400 m, 1232 m, 1145 w, 1078 vs, 1028 s, 902 m, 812 m, 692 w.

**H_3_O[Tb(Q**^cy^**)_4_], 4.** Colorless plates, 73%. Anal. Calc. for: C_68_H_79_N_8_O_9_Tb: C, 62.28; H, 6.07; N, 8.55; Tb, 12.12%. Found: C, 62.5; H, 6.2; N, 8.6; Tb, 11.7. IR (cm^−1^): 3391 m (ν O–H^H3O+^), 2929 m, 2852 m (ν C-H^Cyclohexyl^), 1661 w (δ O–H^H3O+^), 1616 m (ν C=O^Diketone^), 1593 m, 1584 m, 1500 m (ν C–C), 1400 m, 1230 m, 1145 w, 1078 vs, 1028 s, 902 m, 812 m, 692 w.

**H_3_O[Dy(Q**^cy^**)_4_]**, **5**. Pale yellow plates, 70%. Anal. Calc. for C_68_H_79_N_8_O_9_Dy: C, 62.11; H, 6.06; N, 8.52; Dy, 12.36%. Found: C, 61.9; H, 6.1; N, 7.9%. IR (cm^−1^): 3390 m (ν O–H^H3O+^), 2929 m, 2852 m (ν C-H^Cyclohexyl^), 1660 w (δ O–H^H3O+^), 1616 m (ν C=O^Diketone^), 1594 m, 1584 m, 1500 m (ν C–C), 1400 m, 1230 m, 1153 w, 1078 vs, 1028 s, 902 m, 812 m, 692w.

The silver complexes [AgLn(Q^cy^)_4_]_n_ **6–10** were obtained according to the following general procedure given in detail for [AgSm(Q^cy^)_4_]_n_.

**[AgSm(Q**^cy^**)_4_]_n_**, **6**. 0.069 mg (0.222 mmol) of a SmCl_3_^.^6H_2_O and 1.5 mL of H_2_O were placed in a centrifugation tube. Then, 0.100 mL of a NH_3_ solution ~12 M were added, the volume was adjusted to 2 mL with water and centrifuged (8000 rpm, 5 min). The precipitate was carefully washed with water (4 × 2 mL), 96% ethanol (2 × 2 mL) and added to a solution of 0.284 g (1.00 mmol) of HQ^cy^ in 20 mL of 96% ethanol at room temperature. The mixture was stirred without heating until the precipitation of the formed lanthanide hydroxide. Then, 0.224 mL of a 10% solution of triethylamine (0.222 mmol) in 96% ethanol was added dropwise and the solution was filtered through a 0.25 μm PTFE syringe membrane filter. Subsequent operations were performed in the dark.

A solution of 0.0377 g (0.222 mmol) silver nitrate in 5 mL of acetonitrile was added dropwise at a rate of 1 mL per minute. An abundant precipitate begins to form already when the first drops are added. The suspension was stirred at room temperature for another 15 min, after which it was filtered on a glass porous filter, washed with ethanol (2 × 5 mL), acetonitrile (2 × 5 mL) and diethyl ether (2 × 5 mL). The precipitate was dried in a darkened desiccator over P_4_O_10_ for 48 h.

**[AgSm(Q**^cy^**)_4_]_n_**, **6**. Pale yellow powder, 82%. Anal. Calc. for C_68_H_76_N_8_O_8_AgSm: C, 58.69; H, 5.50; N, 8.05; Ag, 7.75; Sm, 10.80%. Found: C, 58.2; H, 5.6; N, 8.0; Ag, 7.5; Sm, 11.3%. IR (cm^−1^): 3058 m, 2929 m, 2852 m(ν C-H^Cyclohexyl^), 1627 vs (ν C=O^Diketone^), 1600 m, 1500 s, 1434 w, 1395 w, 1370 w, 1080 m, 1035 vw, 1010 vw, 979 m, 812 vw, 758 w, 693 w, 622 w, 452 w.

**[AgEu(Q**^cy^**)_4_]_n_**, **7**. White powder, 83%. Anal. Calc. for C_68_H_76_N_8_O_8_AgEu: C, 58.62; H, 5.50; N, 8.04; Ag, 7.74; Eu, 10.91%. Found: C, 58.9; H, 5.5; N, 8.2; Ag, 7.6; Eu, 10.8%. IR (cm^−1^): 3060 m, 2929 m, 2852 m(ν C-H^Cyclohexyl^), 1628 vs (ν C=O^Diketone^), 1599 m, 1500 s, 1435 w, 1392 w, 1377 vw, 1080 m, 1010 w, 979 m, 756 w, 691 w, 623 w, 450 w.

**[AgGd(Q**^cy^**)_4_]_n_**, **8**. White powder, 74%. Anal. Calc. for C_68_H_76_N_8_O_8_AgGd: C, 58.40; H, 5.48; N, 8.01; Ag, 7.71; Gd, 11.24%. Found: C, 58.2; H, 5.8; N, 8.0; Ag, 7.7; Gd, 11.4%. IR (cm^−1^): 3061 m, 2929 m, 2852 m (ν C-H^Cyclohexyl^), 1628 vs(ν C=O^Diketone^), 1598 m, 1500 s, 1436 w, 1392 w, 1372 vw, 1080 m, 1010 w, 978 m, 756 w, 690 w, 623 w, 448 vw.

**[AgTb(Q**^cy^**)_4_]_n_**, **9**. White powder, 82%. Anal. Calc. for C_68_H_76_N_8_O_8_AgTb: C, 58.33; H, 5.47; N, 8.00; Ag, 7.70; Tb, 11.35%. Found: C, 58.4; H, 5.6; N, 8.2; Ag, 7.7; Tb, 11.4%. IR (cm^−1^): 3070 m, 2928 m, 2852 m, (ν C-H^Cyclohexyl^) 1631 vs(ν C=O^Diketone^), 1596 m, 1501 s, 1543, 1435 w, 1371 w, 1080 m, 979 m, 902 vw, 756 w, 691 w, 622 w, 449 w.

**[AgDy(Q**^cy^**)_4_]_n_**, **10**. White powder, 77%. Anal. Calc. for C_68_H_76_N_8_O_8_AgDy: C, 58.18; H, 5.46; N, 7.98; Ag, 7.68; Dy, 11.58%. Found: C, 58.6; H, 5.2; N, 7.9; Ag, 7.8; Dy, 11.2%. IR (cm^−1^): 3071 m, 2929 m, 2852 m (ν C-H^Cyclohexyl^), 1631 vs(ν C=O^Diketone^), 1595 m, 1502 s, 1542, 1435 w, 1371 w, 1081 m, 979 m, 692 w, 622 w, 449 w.

Single crystals of [AgGd(Q^cy^)_4_]_n_ (**8**) were obtained by suspending 0.05 g of the powder in 10 mL of acetonitrile at room temperature in the dark. The precipitate was then separated by centrifugation (8000 rpm), and the solution was filtered through a 0.25 μm PTFE syringe membrane filter. Slow evaporation of the solution at room temperature allowed the obtaining of small single crystals of **8** suitable for investigations on the synchrotron equipment. The isostructurality of other compounds **6**, **7**, **9** and **10** was confirmed by powder diffraction methods (see SI, Figure S2).

### 2.2. Apparatus

Elemental analyses were performed with an Elemental Vario MicroCube CHNO(S) (Elementar Americas Inc., Ronkonkoma, NY, USA) analyser. The Ln^3+^ content was determined by complexometric titration with a Trilon B solution in the presence of Xylenol Orange as an indicator [77]. The silver content was determined by titration with standard KSCN solution using Fe^3+^ to indicate the end point [78]. Before the analysis, the complexes were decomposed by heating with concentrated HNO_3_.

Absorption spectra for all the complexes were obtained on a JASCO V-770 (Jasco, Tokyo, Japan) spectrophotometer operating within 200–3200 nm. Concentrations of the solutions were approximately 10^−5^ M. For solutions, the measurements were performed using quartz cells with a 1 cm pathlength. 

IR spectra were registered in the range 4000–400 cm^−1^ in KBr pellets using a Perkin-Elmer system Spectrum One 100 FTIR spectrometer (PerkinElmer, Inc., Waltham, MA, USA).

The X-ray diffraction data sets for crystals of H_3_O[Ln(Q^cy^)_4_] (**1**: Ln = Sm; **2**: Ln = Eu; **3**: Ln = Gd; **4**: Ln = Tb) were collected on a Bruker SMART APEX II (Bruker, Billerica, MA, USA) diffractometer equipped with a CCD detector (Mo-K_α_, *λ* = 0.71073 Å, graphite monochromator) [79]. X-ray diffraction data for [AgGd(Q^cy^)_4_]_n_ **8** were collected on the ‘Belok’ beamline of the Kurchatov Synchrotron Radiation Source (National Research Center ‘Kurchatov Institute’, Moscow, Russia) in *φ*-scan mode with 1° step (*λ* = 0.79312 Å) using a Rayonix SX165 (Rayonix, L.L.C., Evanston, IL, USA) CCD detector [80]. The structures were solved by direct methods and refined by the full-matrix least squares in the anisotropic approximation for non-hydrogen atoms. The calculations were carried out by the SHELX-2014/2018 program package [81] using Olex2 1.2 [82]. The hydrogen atoms of the ligands were positioned geometrically and refined using the riding model. Solvent molecules in [AgGd(Q^cy^)_4_]_n_ **8** which could not be localized were removed by the SQUEEZE procedure [83]. The crystallographic parameters for investigated crystals and the structure refinement details are given in Table 1. Crystallographic data for structures reported in this paper have been deposited with the Cambridge Crystallographic Data Center (H_3_O[Eu(Q^cy^)_4_]—2233167, H_3_O[Gd(Q^cy^)_4_]—2233168, H_3_O[Tb(Q^cy^)_4_]—2233169, [AgGd(Q^cy^)_4_]_n_—2233170). 

Photoluminescence and excitation spectra in the visible region for all the complexes were measured in solid state using a Horiba Jobin-Yvon Fluorolog QM-75-22-C spectrofluorimeter with an installed 75 W xenon arc lamp (PowerArc, HORIBA, Ltd., Kyoto, Japan). A Hamamatsu R13456 cooled photomultiplier tube sensitive in the UV-Vis-NIR region (200–950 nm) was used as the detector. For the NIR spectral region measurements the same setup was used, except for the detector, which was replaced by a Hamamatsu H10330 (Hamamatsu Photonics, Hamamatsu, Japan) cooled photomultiplier tube sensitive in the NIR region (950–1700 nm). Photoluminescent decays in the visible region were recorded for all the complexes in solid state on the same device; however, the excitation source was changed to a pulsed xenon lamp with a 50 μs pulse duration and 100 Hz repetition rate. 

Photoluminescence quantum yields were obtained for solid samples by the absolute method with the use of the same experimental setup, equipped by integration sphere G8 (GMP, Renens, Switzerland).

For all optical measurements, the corresponding instrument response functions were taken into account. The experiments were performed in air at atmospheric pressure. Degradation of the optical properties was not observed during the experiments. Commercially available reagents and solvents (Sigma-Aldrich, Darmstadt, Germany) were used as received. Ln^3+^ chlorides were obtained by dissolution of the corresponding oxides (99.999%, LANHIT, Moscow, Russia) in concentrated hydrochloric acid (reagent grade, XPC). Ligand HQ^cy^ was prepared according to the published method [37].

## 3. Results

### 3.1. Synthesis

The synthesis of complex tetrakis acids H_3_O[Ln(Q^cy^)_4_] was carried out according to the general procedure proposed by us for a dysprosium derivative [57]. Temperature control is important to avoid formation of tris complexes [Ln(Q^cy^)_3_(H_2_O)] [57,84]. Polymer complexes can be obtained by the reaction of silver (I) nitrate with a solution of H_3_O[Ln(Q^cy^)_4_] acids, but the yield of the reaction somewhat increases after its preliminary neutralization with the use of triethylamine. The complexes gradually darken in the light, so all operations must be performed in subdued light. The polymer structure makes [AgLn(Q^cy^)_4_]*_n_* complexes extremely poorly soluble in most common solvents. The use of DMF for recrystallization is impossible, since it is accompanied by the decomposition of the complex with the formation of a silver mirror on the walls of the vessel. DMSO also causes decomposition of the complexes with the formation of an unspecified black precipitate (possibly silver sulfide). Among other tested solvents (methanol, ethanol, acetone, chloroform, ethyl acetate), only acetonitrile can be a possible medium for recrystallization. Attempts to grow large crystals under solvothermal conditions were unsuccessful, and only small crystals of the gadolinium complex were studied at the synchrotron facility. However, IR spectroscopy, elemental analysis, and powder diffraction data confirm that all [AgLn(Q^cy^)_4_]*_n_* complexes are isomorphic to the gadolinium polymeric complex [AgGd(Q^cy^)_4_]*_n_*.

### 3.2. Single Crystal Structures

**Figure 1:**
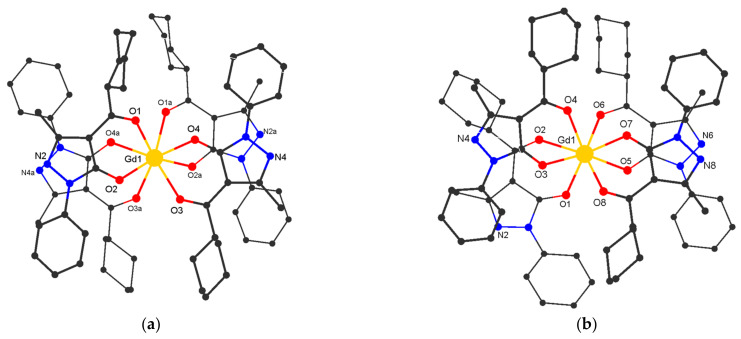
Structure of the anionic moiety of **[Gd(Q**^cy^**)_4_]**^−^ in **H_3_O[Gd(Q**^cy^**)_4_] 3** (**a**) and in **[AgGd(Q**^cy^**)_4_]_n_ 8** (**b**) (H atoms are omitted).

**Figure 2:**
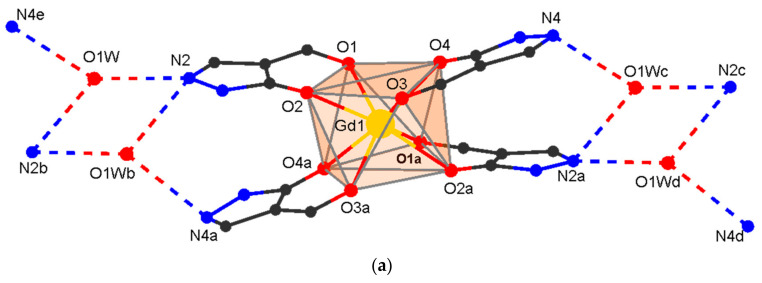

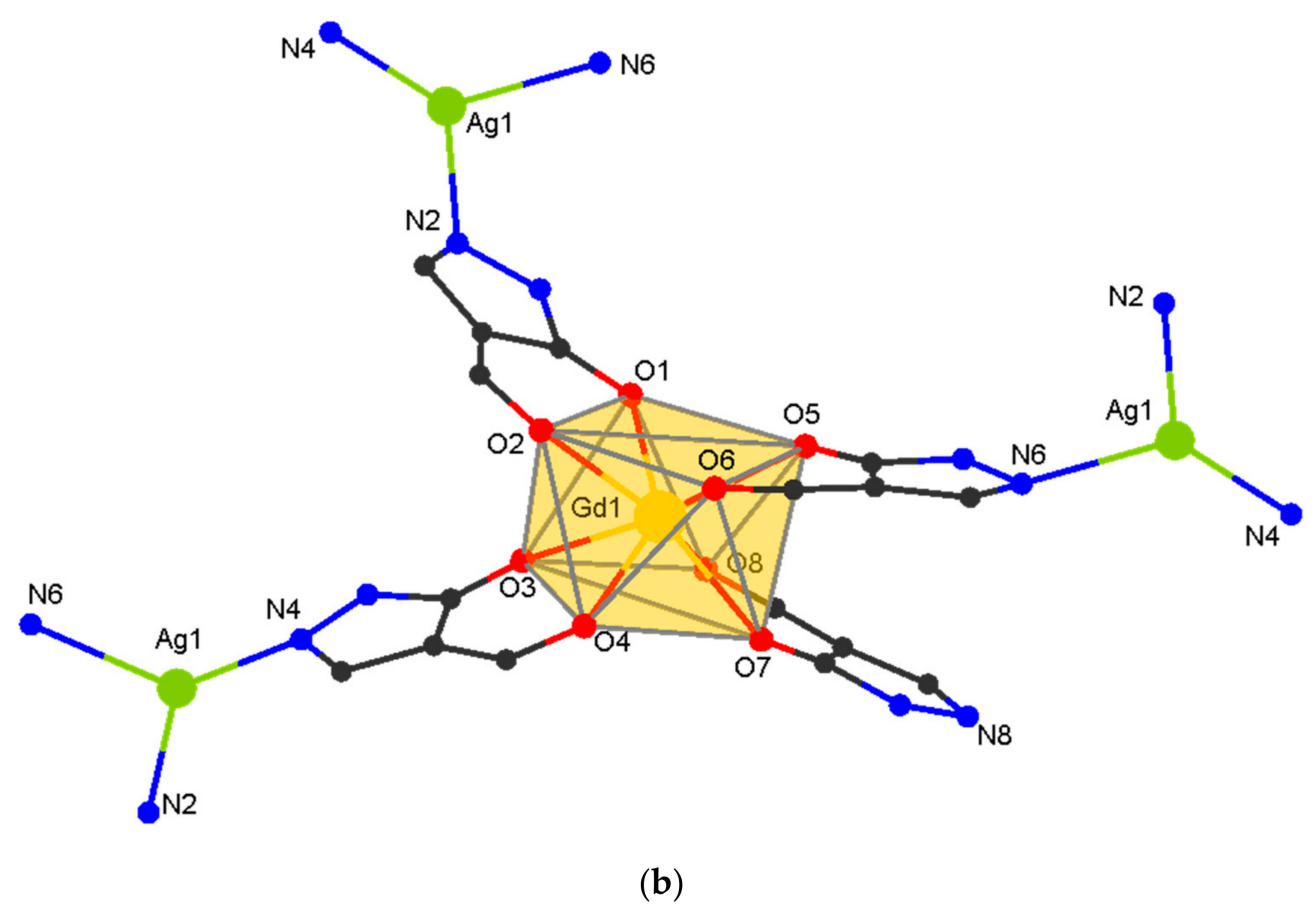
Fragment of the crystal packing in H_3_O[Gd(Q^cy^)_4_] **3** (**a**) and [AgGd(Q^cy^)_4_]*_n_* **8** (**b**) (phenyl, cyclohexyl and methyl groups are omitted for clarity).

Complexes [H_3_O][Ln(Q^cy^)_4_] **1–4** (**1**: Ln = Sm; **2**: Ln = Eu; **3**: Ln = Gd; **4**: Ln = Tb) crystallize in the monoclinic space group C2/c (for crystals with Ln = Sm, only cell parameters were determined; Table 1) and are isostructural to the previously described dysprosium complex [H_3_O][Dy(Q^cy^)_4_] **5** [57]. These compounds consist of the [Ln(Q^cy^)_4_]^−^ anion and of the outer sphere H_3_O^+^ (Figure 1a). The Ln atom is coordinated by four chelating Q^cy^ ligands to form a LnO_8_ polyhedron in a square antiprismatic geometry (main distances and angles are given in Table 2). The oxygen atom of H_3_O^+^ is disordered in two positions (a two-fold axis passes between them) and form H-bonds with two N atoms of one complex molecule and one N atom of the neighboring molecule (Figure 2a; Table S1). Additionally, the molecule is stabilized by intramolecular C-H…O/N as well as C-H…π interactions (Tables S1 and S2). In the crystal, π-π intermolecular interactions occur between the pyrazole rings of neighboring molecules (Table S3). As in the other described H(H_2_O)_n_[LnQ_4_] tetrakis-acids, stabilization is achieved by strong intermolecular hydrogen bonds [57,67,68,69]. H-bonding between the anionic fragments [**Ln(Q**^cy^**)_4_**]^−^ leads to the formation of 1D supramolecular chains. 

**Figure 3:**
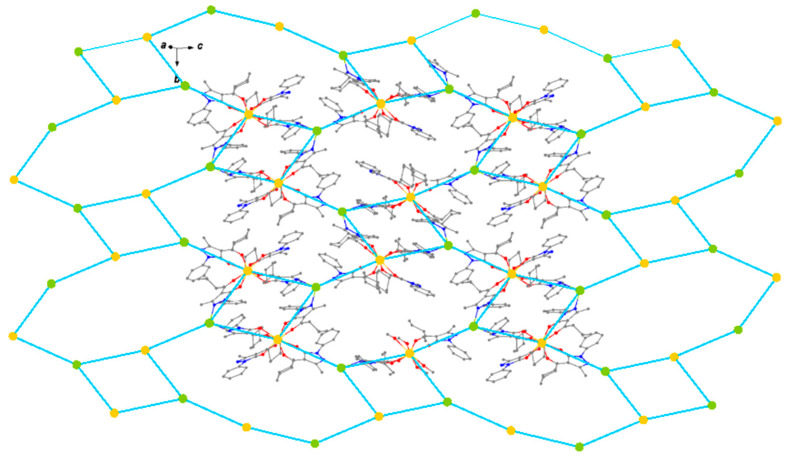
Crystal packing of [AgGd(Q^cy^)_4_]_n_ **8**.

[AgGd(Q^cy^)_4_]_n_ **8** crystallizes in the monoclinic space group P2_1_/n. It contains the fragment [Gd(Q^cy^)_4_] and the Ag center. The structure of the [Gd(Q^cy^)_4_]^−^ fragment in **8** is similar to that in **3** (Figure 1b), the geometry of the metal atoms environment (GdO_8_) being slightly distorted (Figure 2, Table 2). The molecule is stabilized by intramolecular C-H…O/N interactions (Table S1). Each Ag atom is coordinated by three pyridinic N atoms of the pyrazole rings from three [Gd(Q^cy^)_4_] fragments. Each [Gd(Q^cy^)_4_] fragment is bonded to three Ag atoms; thus, three of the four pyrazole rings are involved in formation of the polymeric structure (Figure 2b). Binding of [Gd(Q^cy^)_4_]^−^ to Ag^+^ leads to the formation of a layered structure with a well-known topological type **fes** [85], where both structural building units are 3-coordinated nodes (Figure 3). In the crystal, π-π intermolecular interactions occur between the pyrazole and the phenyl rings of neighboring anionic framents (Table S3). The minimum interatomic distance between successive Gd atoms in the layer is 8.788 Å, and that of neighboring layers is 13.040 Å. The interlayer space is filled by cyclohexyl substituents.

### 3.3. Optical Properties

**Figure 4:**
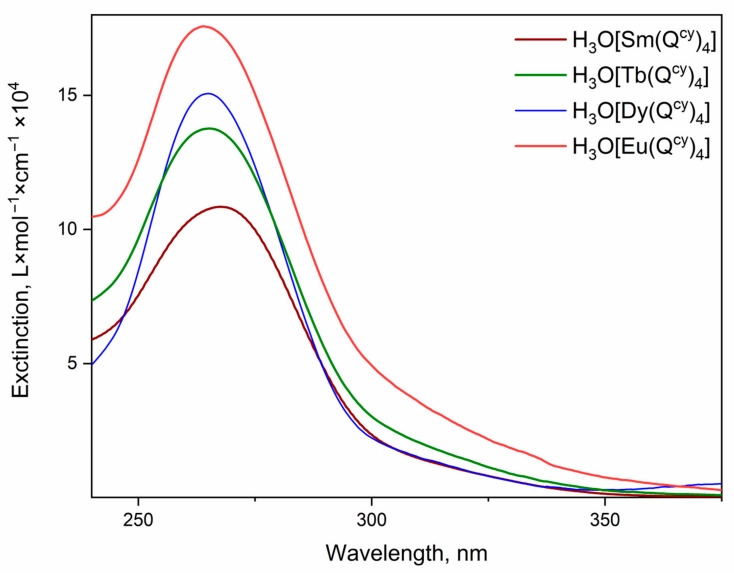
UV-VIS spectra of [H_3_O][Ln(Q^cy^)_4_] complexes.

The absorption spectra of H_3_O[Ln(Q^cy^)_4_] **1–4** dissolved in MeCN are shown in Figure 4. All complexes **1–4** reveal intensive absorption in the range 250–375 nm. The observed absorption maximum at 265 nm is originated from the π-π* transition in the β-diketone moiety [57]. The spectra qualitatively resemble each other; therefore, despite the variation of the central ion, the ligand environment energy structure remains the same. Moreover, the coordination of the central ion by the ligands allows an increase in the molar extinction of the complexes up to 10^5^ L × mol^−1^ × cm^−1^, with respect to the low values obtained for the free ions (~10 L × mol^−1^ × cm^−1^). Notably, we did not observe any absorption bands related to ion transitions due to a high ligand absorption rate.

The energy of the first excited singlet state (S_1_) was estimated using the tangent method [26] for the absorption spectra. For all the complexes, the S_1_ energies are nearly equal and are 27,300 cm^−1^. Due to the low solubility of [AgLn(Q^cy^)_4_]_n_ **6–10** in most of the solvents generally employed (acetonitrile, methanol, dichloromethane), it is impossible to record their absorption spectra. For this reason, diffuse reflectance spectra were studied (see below).

**Figure 5:**
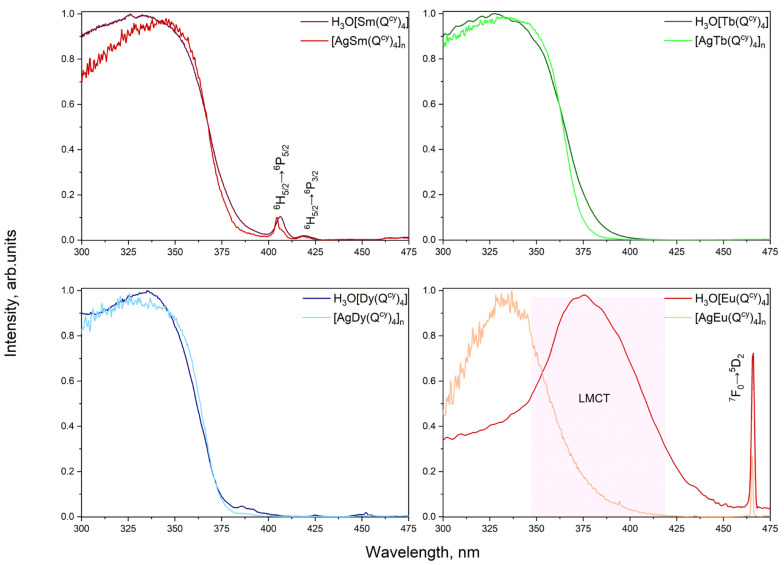
PL excitation spectra for all the complexes pairwise.

**Figure 6:**
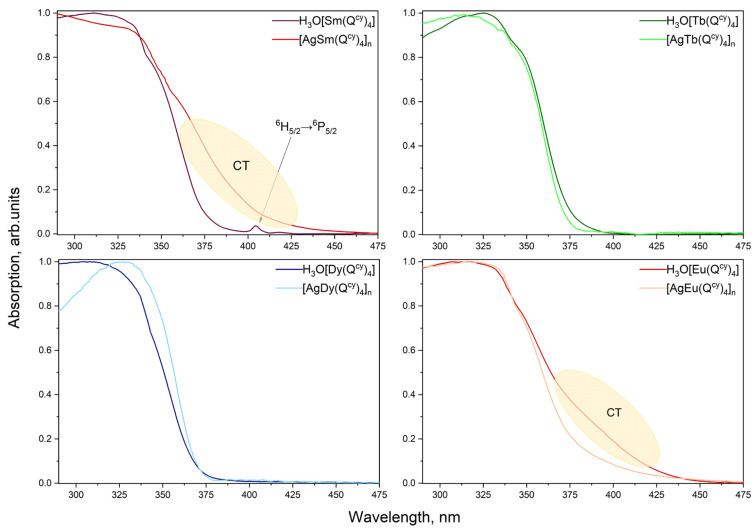
Diffuse reflection spectra for all the complexes pairwise.

PL excitation spectra, recorded for all the complexes in the solid state, are shown in Figure 5. Apart from H_3_O[Eu(Q^cy^)_4_], all the other complexes reveal the strong ion-centered luminescence under the optical excitation in a wide spectral range from 285 to 425 nm. The wide excitation band with the maximum at 340 nm is specific of the sensitization of the luminescence through the π*-π transition in the β-diketone moiety. However, the spectrum of the H_3_O[Eu(Q^cy^)_4_] shows a maximum at 380 nm with the FWHM (full width at half maximum) of 75 nm. We suggest that this band is related to the excitation due to ligand-to-metal charge-transfer (LMCT) [86]. Actually, the long wavelength shoulder observed for **2** in the diffuse reflection spectrum (see Figure 6) can result from different charge transfer processes. No LMCT with such energies has been observed for **4** due to the high redox potential of terbium ion. As the spectrum of **4** reveals no absorption within 360–450 nm, the shoulder in **2** can be associated to LMCT.

Interestingly, the spectra of complexes containing Ag^+^ are qualitatively similar, while the diffuse reflection spectra (see Figure 6) of [AgSm(Q^cy^)_4_]_n_ **6** and [AgEu(Q^cy^)_4_]_n_ **7** also reveal charge transfer processes. 

The weak narrow bands observed in the excitation spectra of **1** and **2** and of **6** and **7** are related to the H5/24→P5/26 and H5/24→P3/26 transitions of the Sm^3+^ ion and the F07→D25 transition of the Eu^3+^ ion. Such behavior implicitly proves effective energy transfer of electronic excitation from donor-ligand to acceptors-ions.

**Figure 7:**
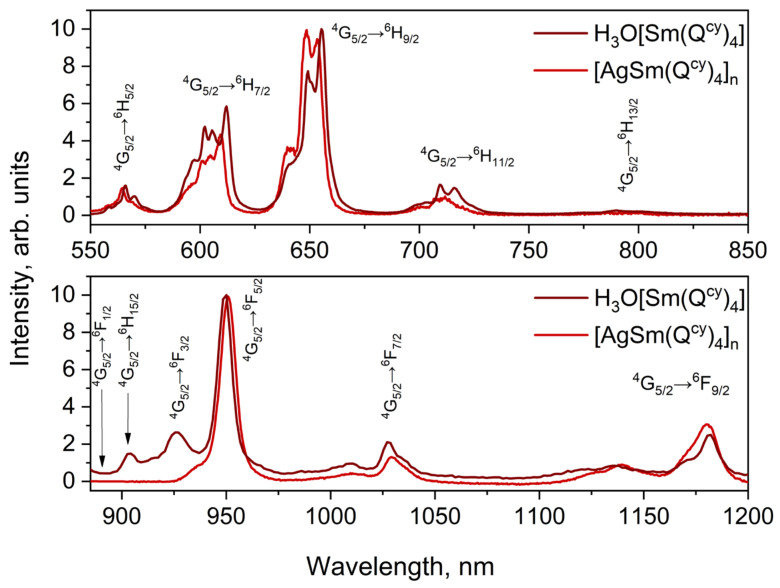
Photoluminescence spectra of Sm^3+^ complexes **1** and **6**.

**Figure 8:**
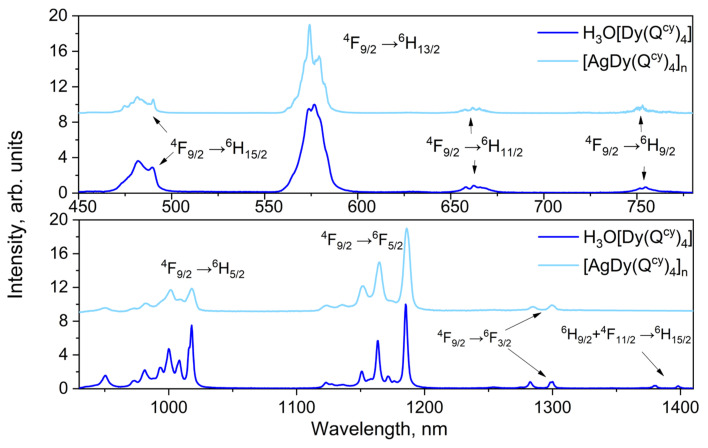
Photoluminescence spectra for Dy^3+^ complexes **5** and **10**.

**Figure 9:**
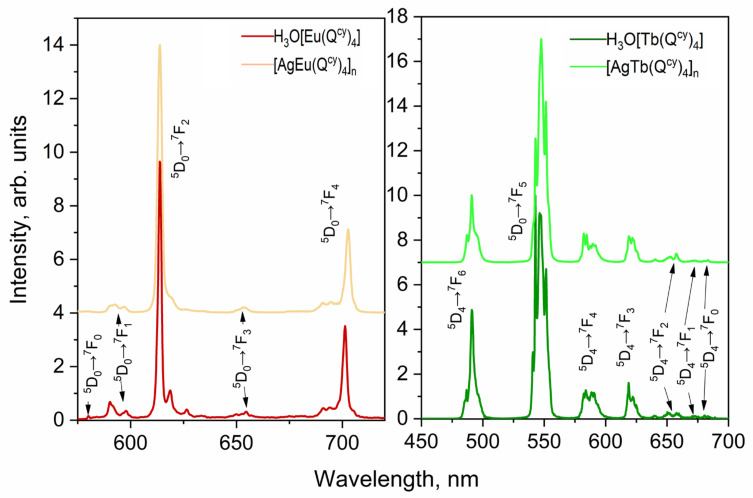
Photoluminescence spectra for Eu^3+^ (**2** and **7**) and Tb^3+^ (**4** and **9**) complexes.

Intensive luminescence of all the investigated complexes is observed under optical excitation via absorption bands associated with ligand environment (see Figure 7, Figure 8 and Figure 9). In all the ionic species, we observed the narrow spectral bands typical of f*-f transitions (see Table S5). The correlation of the emission bands with the f*-f transitions was performed according to [57,87,88]. Notably, we observed no ligand emission in the emission spectra, which also indicates the relatively effective energy transfer of electronic excitation from ligand to ions [26].

The luminescence spectra of the complexes containing H_3_O^+^ and Ag^+^ have no significant differences pairwise in Stark splitting and emission band positions. Thereby, the replacement of H_3_O^+^ with the Ag^+^ produces no change in the symmetry of coordination polyhedral [26,89,90]. 

PL decays were recorded under the excitation wavelengths corresponding to maxima of excitation spectra with the registration at the photoluminescence maxima (see Figures S3 and S4). However, all the decay curves were estimated by a biexponential function. The obtained characteristic lifetimes are presented in Table 3. The replacement of H_3_O^+^ with Ag^+^ in the ligand environment leads to an increase of observed lifetimes for all the complexes except for Tb^3+^ ion complexes. Photoluminescence overall quantum yield (PLQY, Ф) values recorded under UV excitation are presented in Table 3.

To determine the triplet level energy of the ligand in lanthanide complexes, measurements of the low-temperature phosphorescence spectra of Gd^3+^ derivatives are usually used [26,91]. The combination of the high energy of the natural resonance level (>30,000 cm^−1^) of Gd^3+^ ions, the paramagnetic nature and the heavy atom effect contribute to the fact that the low-temperature emission spectra of gadolinium complexes mainly contain a phosphorescence transition whose energy corresponds to the triplet level value [92].

**Figure 10:**
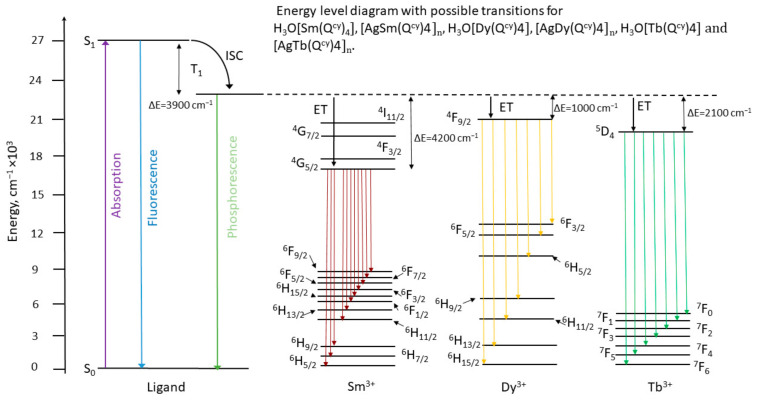
Energy diagram with possible transitions for **1** and **6** (Sm^3+^), **5** and **10** (Dy^3+^), **4** and **9** (Tb^3+^).

**Figure 11:**
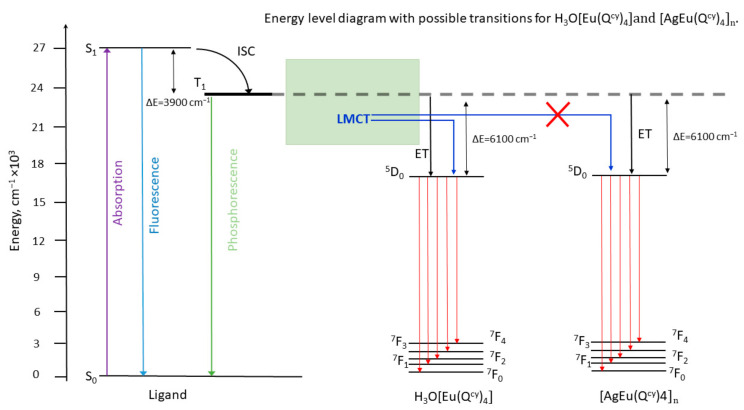
Energy diagram with possible transitions for [H_3_O][Eu(Q^cy^)_4_] **2** and [AgEu(Q^cy^)_4_]_n_ **7**.

The phosphorescence spectra measured at 77 K for the solid state Gd^3+^ complexes are shown in Figure S5. The energy of the first excited triplet state was derived from the maximum of the fitting component corresponding to the zero-phonon line in the spectrum, according to the well-known procedure [93]. Thus, based on the spectral and kinetic measurements, we were able to construct the Jablonsky–Crossby energy diagrams for complexes reported here (Figure 10 and Figure 11). The luminescence characteristics of the europium complexes are caused by the influence of the LMCT state, which quenches the luminescence in the H_3_O[Eu(Q^cy^)_4_] complex and is associated with the ease ($E_{Eu^{3+}/Eu^{2+}}^{0}=-0.35\;V)$ [86] reduction of the europium ion. The high oxidation potential ($E_{Ag^{+}/Ag}^{0}=0.799\;V)$ of silver blocks this mechanism, which makes it possible to improve the luminescence characteristics of europium complexes. We believe that the low luminescence efficiency of other previously described europium acylpyrazolonates can also be explained by LMCT quenching [37,65]. 

The replacement of H_3_O^+^ with Ag^+^ in the ligand environment results in a significant decrease of PLQY, except for the Eu^3+^ complexes. The distance between the ions in the crystal package decreases practically two times after the replacement of H_3_O^+^ with Ag^+^, which results in cross-relaxation processes between the ions, which suppresses the radiative relaxation. Surprisingly, the Eu^3+^ complexes have low values of quantum yield; in particular, the Ф of [AgEu(Q^cy^)_4_]_n_ is 0.3% and H_3_O[Eu(Q^cy^)_4_] PLQY is lower than 0.005%. 

All the complexes have rather low extinction coefficients for the absorption bands characteristic of the lanthanide ions (see Figure S6). However, we successfully performed the Judd-Ofelt analysis [94,95] for the H_3_O[Sm(Q^cy^)_4_] complex to estimate the τ_rad_ radiative lifetime value for the ^4^G_5/2_ emission state of the Sm^3+^ ion. The general procedure was the same as reported in [87]. It should be noted that for minimizing the root-mean-square deviation (RMS) in our calculations we include only transitions within the energy range 8000–22,000 cm^−1^; see Table 4. The oscillator strengths of the electric dipole transitions determined from the optical absorption spectrum and calculated ones, Ω_t_ (t = 2,4,6) Judd–Ofelt intensity parameters and radiative rate of the emission state of Sm^3+^ ion in our complex H_3_O[Sm(Q^cy^)_4_] **1** are presented in Table 4. The refractive index value 1.47 was used in our calculations according to [96]. Theoretical branching ratios for luminescent transitions are in good agreement with the experimental data and the low RMS value allow us to consider our results to be accurate; see Table 5. 

The evaluated τ_rad_ value of ca. 3.2 ms is of the same order of magnitude of those reported for other Sm^3+^ complexes with similar chemical environments [87,97]. Measured luminescence decay times, obtained for the resonant excitation of the Sm^3+^ ion in the complex at 405 nm, are slightly higher than those presented in Table 5, and could also be fitted by a biexponential function with τ_1_ = 49.1 ± 0.8 μs and τ_2_ = 80.1 ± 1.4 μs. The estimated internal quantum yield of the complex is near 2% (1.5–2.4%, with respect to the biexponential luminescence decay behavior and accuracy of the calculations); the estimated and measured values are very close, and the sensibilization coefficient is close to 1. 

**Figure 12:**
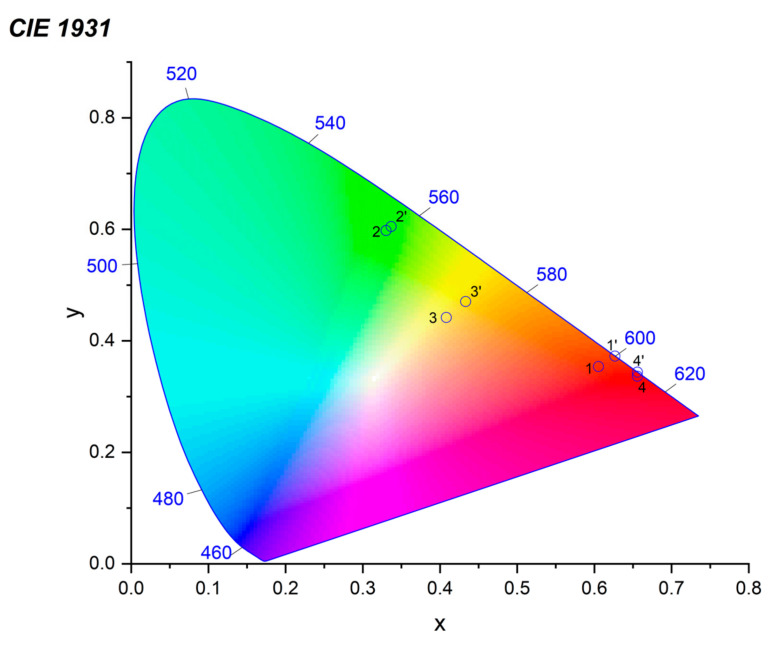
CIE diagram for complexes **1, 6, 2, 7, 3, 8, 4** and **9**.

The complexes reported in this work brightly emit in red, yellow and green spectral regions. For each compound, color diagrams were made (see Figure 12). The color coordinates for all the complexes are presented in Table S6.

The internal quantum yield can be estimated by the following formula

$$PLQY_{in}=\frac{k_{rad}}{k_{rad}+k_{nrad}}.$$

Since the rate constant of the magnetic-dipole transition ^5^D_0_ →^7^F_1_ in Eu^3+^, $k_{MD}$= 14.65 s^−1^, does not depend on the electric field induced by the ligand, the value of $k_{rad}$ can be determined by the relationship:

$$k_{rad}=k_{MD}n^{3}\frac{I_{tot}}{I_{MD}},$$

where *n* is the refractive index and $\frac{I_{tot}}{I_{MD}}$ is the ratio between the total integral luminescence intensity and the integral intensity of the magnetic-dipole transition. The $k_{nrad}$ constant was estimated using a simple dependence:

$$k_{nrad}=\frac{1}{\tau_{obs}}-k_{rad}\;$$

comprising the calculated $k_{rad}$ and the observed attenuation time $\tau_{obs}$ measured with resonant excitation of Eu^3+^ at a wavelength of 464 nm. The calculated internal quantum yields $PLQY_{in}$, the total quantum yields *PLQY* measured by the absolute method, and the sensitization coefficients η are presented in Table 6.

## 4. Conclusions

The synthesis of coordination polymers based on lanthanides acylpyrazolonates due to the formation of a heterometallic *4d-4f* complexes is shown for the first time. The resulting coordination polymers exhibit bright luminescence caused by *f-f* transitions of the central ions. The change from a monomeric to a polymeric structure leads to various changes in the luminescence characteristics: while for samarium, terbium, and dysprosium derivatives the luminescence efficiency somewhat decreases, in the case of europium, on the contrary, it increases significantly. This phenomenon is due to the special role of LMCT states in europium acylpyrazolonates.

## Figures and Tables

**Table 1:** The main crystallography data and refinement details for structures H_3_O[Ln(Q^cy^)_4_] (**1–4**) and [AgGd(Q^cy^)_4_]_n_ **8**.

Complex/Parameters	1 ^a^	2	3	4	8
**Empirical formula**		C_68_H_79_EuN_8_O_9_	C_68_H_79_GdN_8_O_9_	C_68_H_79_N_8_O_9_Tb	C_68_H_76_AgGdN_8_O_8_
**Formula weight**		1304.35	1309.64	1311.31	1398.48
**Crystal system**	Monoclinic				
**Space group**	*C*	*C*2/*c*			*P*2_1_/*n*
***T*, K**	296	296	150	150	100
***a*, Å**	14.907 (8)	14.8703 (6)	14.8096 (5)	14.7960 (5)	15.0083 (11)
***b*, Å**	25.244 (16)	25.2111 (14)	25.1474 (9)	25.1641 (11)	17.7224 (16)
***c*, Å**	16.773 (10)	16.9977 (11)	16.7390 (9)	16.7208 (9)	25.1710 (19)
***β*, deg.**	93.87 (2)	94.7700 (10)	94.1050 (10)	94.3260 (10)	92.623 (3)
***V*, Å^3^**	6297 (2)	6350.3 (6)	6218.0 (5)	6207.9 (5)	6688.0 (9)
**Z**		4	4	4	4
***D*_calc_, g cm^3^**		1.364	1.399	1.403	1.389
***μ*, mm ^−1^**		1.051	1.131	1.204	1.782
**θ_max_, deg.**		27.10	30.52	26.61	29.716
**Index ranges**		−18 ≤ *h* ≤ 19 −32 ≤ *k* ≤ 32 −19 ≤ *l* ≤ 21	−20 ≤ *h* ≤ 21 −35 ≤ *k* ≤ 35 −23 ≤ *l* ≤ 23	−18 ≤ *h* ≤ 18 −31 ≤ *k* ≤ 31 −19 ≤ *l* ≤ 21	−18 ≤ *h* ≤ 18 −22 ≤ *k* ≤ 22 −31 ≤ *l* ≤ 31
***F* (000)**		2712	2716	2720	2860
***R*_int_**		0.1612	0.1319	0.0697	0.2473
**Number of collected/total reflections**		34,077/7002	37,516/9476	28,718/6414	60,971/13,654
**Number of reflections (*I* > 2*σ*(*I*))**		5265	6920	5570	4961
**Number of parameters**		393	395	395	779
***GooF***		1.060	1.077	1.095	0.881
***R*_1_, *wR*_2_ (*I* > 2*σ*(*I*))**		0.0699, 0.1542	0.0875, 0.1535	0.0467, 0.0980	0.0911/0.1939
**Δ*ρ*_max_, *ρ*_min_(e/Å^3^)**		0.895, −1.774	5.325, −2.815	1.467, −1.238	0.783/−2.114

^a^ Only cell parameters were determined.

**Table 2:** Selected bond lengths, shortest interatomic distances (Å), angles (deg.) and symmetry of polyhedron LnO_8_ in H_3_O[Ln(Q^cy^)_4_] **2–4** (Ln = Eu, Gd, Tb) and [AgGd(Q^cy^)_4_]_n_ **8**.

Complex/Parameter	2	3	4	8
Ln-O	2.354 (4)–2.436 (4)	2.349 (4)–2.414 (4)	2.328 (3)–2.412 (3)	2.287 (8)–2.448 (9)
O-Ln-O (1,3-diketone)	71.14 (13), 72.13 (14)	71.75 (13), 72.28 (14)	72.22 (9), 72.66 (9)	68.1 (3)–70.8 (3)
Ag-N	-	-	-	2.229 (11)–2.403 (9)
N-Ag-N	-	-	-	99.4 (4), 129.3 (4), 131.2 (4)
Symmetry of the LnO_8_ polyhedron with *S*_Q_ (*p*) value ^a^	Square antiprism, *D*_4d_, 0.322	Square antiprism, *D*_4d_, 0.254	Square antiprism, *D*_4d_, 0.246	Square antiprism, *D*_4d_, 0.322

^a^ The geometry of the polyhedron corresponds to the symmetry of the analyzed environment; the details of the analysis of the polyhedron are given in Table S4.

**Table 3:** PL quantum yields and PL decays characteristic lifetimes for Sm, Tb, Dy and Eu complexes.

Complex	λ_exc_, nm	PLQY, %	λ_reg_, nm	$\tau_{1},\;\mu s$	$\tau_{2},\;\mu s$
**1**	350	2.0	650	44.1 ± 0.5	73.6 ± 1.2
**6**	350	0.8	650	66.7 ± 3.1	88.2 ± 7.6
**4**	350	55.6	550	593.9 ± 4.8	1403.9 ± 1.9
**9**	350	14.8	550	292.9 ± 2.9	805.7 ± 1.6
**5**	350	2.8 [57]	570	13.0 ± 0.1	42.7 ± 0.1
**10**	350	1.0	570	25.3 ± 1.3	49.8 ± 0.1
**2**	370	n.a.	615	97.9 ± 0.5	486.9 ± 2.0
**7**	350	0.3	615	179.8 ± 2.2	592.5 ± 1.4

**Table 4:** Experimental f_exp_ and calculated f_calc_ oscillator strengths, Judd–Ofelt parameters Ω_t_ (t = 2,4,6), and root-mean-squared deviation RMS for complex H_3_O[Sm(Q^cy^)_4_] **1**.

^6^H_5/2_→^2S+1^L_J_	Wavelength, nm	f_exp_ × 10^8^	f_calc_ × 10^8^
^6^F_7/2_	1237	99.4	99.4
^6^F_9/2_	1086	49.0	49.2
^6^F_11/2_	948	8.5	7.4
^4^F_5/2_	454	1.6	1.8
Ω_2_, cm^2^			8.1 × 10^−20^
Ω_4_, cm^2^			1.8 × 10^−20^
Ω_6_, cm^2^			0.5 × 10^−20^
RMS = 1.3 × 10^−8^			

**Table 5:** Calculated electric-dipole transition probabilities A_rad,_ branching ratios β_calc,_ radiative lifetime τ_rad_ for complex H_3_O[Sm(Q^cy^)_4_].

^4^G_5/2_→^2S+1^L_J_	Wavelength, nm	A_rad_, s^−1^	β_calc_, %
^6^H_5/2_	565	8.65	2.7
^6^H_7/2_	610	46.21	14.9
^6^H_9/2_	650	178.67	57.6
^6^H_11/2_	715	14.27	4.6
^6^H_13/2_	800	0.85	0.27
^6^F_3/2_	936	6.14	1.98
^6^F_5/2_	949	34.99	11.2
^6^F_7/2_	1036	1.47	0.47
^6^F_9/2_	1180	18.67	6.02
τ_rad_ = 3.2 ms; RMS = 1.3 × 10^−8^			

**Table 6:** Photophysical parameters for complexes **2** and **7** in the solid state. Internal quantum yields Φ_in_ and sensitization coefficients η.

Complex	n	I_tot_/I_MD_	k_rad_, s^−1^	k_nrad_, s^−1^	τ_obs_^−1^, s^−1^	PLQY_in_, %	PLQY, %	η
2	1.5	8.87	438	2000	2439	17.90	—	—
7		19.03	941	748	1689	55.70	0.3	0.05385

## Data Availability

Data available upon request.

## Supplementary Materials

The following supporting information can be downloaded at: https://www.mdpi.com/article/10.3390/polym15040867/s1, Figure S1: PXRD patterns of 1–5 and simulated from single crystal data of 2, 3 and 4.; Figure S2: PXRD patterns of 6–10 and simulated from single crystal data of 8; Figure S3: PL decays for the complexes containing H_3_O+; Figure S4: PL decays for the complexes containing Ag^+^; Figure S5: Phosphorescence spectrum of Gd^3+^ complexes at 77K; Figure S6: Absorption spectra for DMSO solution of complex H_3_O[Sm(Q^cy^)_4_] (1) with concentration 3·10^−3^ M; Table S1. D-H…A (D = O, C, A = O, F) interactions in crystals H_3_O[Ln(Q^cy^)_4_] (Ln = Eu, Gd, Tb) and [AgGd(Q^cy^)_4_]*_n_*; Table S2. C-H…π interactions in the crystal [AgGd(Q^cy^)_4_]*_n_*; Table S3. Selected parameters of π-π intermolecular interactions in H_3_O[Ln(Q^cy^)_4_] (Ln = Nd, Sm, Eu, Gd, Tb) and [AgGd(Q^cy^)_4_]*_n_*; Table S4. Continuous Shape Measures (CShM) values [98] for the potential coordination polyhedron of Ln in H_3_O[Ln(Q^cy^)_4_] (Ln = Eu, Gd, Tb) and [AgGd(Q^cy^)_4_]*_n_*; Table S5. Electronic transitions for all the complexes; Table S6. Color coordinates for all the complexes.
