# Supplementary material for: Self-Assembly of a Two-Dimensional Coordination Polymer Based on Silver and Lanthanide Tetrakis-Acylpyrazolonates: An Efficient New Strategy for Suppressing Ligand-to-Metal Charge Transfer Quenching of Europium Luminescence
Source: Polymers (Basel). 2023 Feb 9;15(4):867. doi: 10.3390/polym15040867 (PMC9960442; doi:10.3390/polym15040867)
Supplement: Supplementary file 1 [file polymers-15-00867-s001.zip › polymers-2197833-supplementary.pdf]

Self-Assembly of a Two-Dimensional Coordination Polymer Based on Silver and Lanthanide Tetrakis-Acylpyrazolonates: An Efficient New Strategy for Suppressing Ligand-to-Metal Charge Transfer Quenching of Europium Luminescence

Yuriy A. Belousov [1,2,*], Mikhail T. Metlin [2], Darya A. Metlina [2], Mikhail A. Kiskin [3], Ilya A. Yakushev [3], Trofim A. Polikovskiy [2], Ilya V. Taydakov [2], Andrei A. Drozdov [1], Fabio Marchetti [4] and Claudio Pettinari [5]

[1] Chemistry Department, M.V. Lomonosov Moscow State University, Leninskie Gory Str., Building 1/3, 119991 Moscow, Russia
[2] P. N. Lebedev Physical Institute of Russian Academy of Sciences, Leninsky Prospect 53, 119991 Moscow, Russia
[3] Kurnakov Institute of General and Inorganic Chemistry of the Russian Academy of Sciences, Leninsky Prospekt 31, 119991 Moscow, Russia
[4] School of Science and Tecnology, Chemistry Interdisciplinary Project (ChIP), University of Camerino, Via Madonna delle Carceri, 62032 Camerino, Italy
[5] School of Pharmacy, Chemistry Interdisciplinary Project (ChIP), University of Camerino, Via Madonna delle Carceri, 62032 Camerino, Italy
* Correspondence: belousov@inorg.chem.msu.ru

Contens:

Table S1. D-H…A (D = O, C, A = O, F) interactions in crystals $H_3O[Ln(Q^{cy})_4]$ (Ln = Eu, Gd, Tb) and $[AgGd(Q^{cy})_4]_n$ (the analysis was done using PLATON software).	**2**
Table S2. C-H…π interactions in the crystal $[AgGd(Q^{cy})_4]_n$ (the analysis was done using PLATON software).	**3**
Table S3. Selected parameters of π-π intermolecular interactions in $H_3O[Ln(Q^{cy})_4]$ (Ln = Nd, Sm, Eu, Gd, Tb) and $[AgGd(Q^{cy})_4]_n$ (the analysis was done using PLATON software).	**4**
Table S4. Continuous Shape Measures (CShM) values [98] for the potential coordination polyhedron of Ln in $H_3O[Ln(Q^{cy})_4]$ (Ln = Eu, Gd, Tb) and $[AgGd(Q^{cy})_4]_n$	**5**
Table S5. Electronic transitions for all the complexes.	**6**
Table S6. Color coordinates for all the complexes	**8**
Figure S1. PXRD patterns of 1-5 and simulated from single crystal data of 2, 3 and 4.	**9**
Figure S2. PXRD patterns of 6-10 and simulated from single crystal data of 8.	**10**
Figure S3. PL decays for the complexes containing H_3O^+	**11**
Figure S4. PL decays for the complexes containing Ag^+	**12**
Figure S5. Phosphorescence spectrum of Gd^{3+} complexes at 77K	**13**
Figure S6: Absorption spectra for DMSO solution of complex $H_3O[Sm(Q^{cy})_4]$ (1) with concentration $3\cdot10^{-3}$ M	**14**

Table S1. D-H…A (D = O, C, A = O, F) interactions in crystals $H_3O[Ln(Q^{cy})_4]$ (Ln = Eu, Gd, Tb) and $[AgGd(Q^{cy})_4]_n$ (the analysis was done using PLATON).

Interaction	D-H, Å	H…A, Å	D…A, Å	D-H-A, deg.
**Eu**				
O1W...N2	-	-	2.775(11)	-
O1W...N2 (-*x*,*y*,1/2-*z*)	-	-	2.620(10)	-
O1W...N4 (1-*x*,*y*,1/2-*z*)	-	-	2.844(11)	-
C5-H5...O1	0.93	2.27	2.884(8)	123
C9-H9...N2	0.93	2.48	2.806(9)	101
C9-H9...O1W (-x,y,1/2-z)	0.93	2.40	3.242(12)	151
C22-H22...O3	0.93	2.22	2.866(9)	126
C26-H26...N4	0.93	2.41	2.772(11)	103
C33-H33B...O1 (1-*x*,1-*y*,1-*z*)	0.93	2.58	3.464(9)	151
**Gd**				
O1W...N2	-	-	2.597(9)	-
O1W...N4 (-1+*x*,*y*,*z*)	-	-	2.794(10)	-
O1W...N2 (-*x*,*y*,1/2-*z*)	-	-	2.763(10)	-
C12-H12...O2	0.95	2.26	2.878(6)	122
C16-H16...O1W	0.95	2.33	3.214(11)	155
C16-H16...N2	0.95	2.49	2.817(9)	100
C23-H23A...O2 (1-*x*,1-*y*,1-*z*)	0.95	2.53	3.407(9)	148
C29-H29...O4	0.95	2.23	2.863(8)	123
C33-H33...N4	0.95	2.41	2.773(11)	102
**Tb**				
O1W...N2	-	-	2.592(7)	-
O1W...N4 (-1+*x*,*y*,*z*)	-	-	2.806(7)	-
O1W...N2 (-*x*,*y*,1/2-*z*)	-	-	2.757(7)	-
C13-H13...O1W	0.95	2.34	3.225(7)	154
C13-H13...N2	0.95	2.49	2.823(6)	100
C17-H17...O1	0.95	2.27	2.889(5)	122
C27-H27A...O1	0.95	2.52	3.402(6)	149
C30-H30...N4 O2 (1-*x*,1-*y*,1-*z*)	0.95	2.41	2.769(6)	102
C34-H34...O3	0.95	2.24	2.883(6)	124
**Ag-Gd**				
C5-H5...O1	0.93	2.37	2.833(16)	111
C26-H26...O3	0.93	2.44	2.848(15)	107
C39-H39...O5	0.93	2.52	3.016(16)	114
C56-H56...O7	0.93	2.08	2.77(2)	129
C60-H60...N8	0.93	2.30	2.64(3)	101

Table S2. C-H…π interactions in the crystal [AgGd(Q^{cy})$_4$]$_n$ (the analysis was done using PLATON software).

Interaction	H…Cg, Å	H-Perp, Å	Gamma, deg.	C-H…Cg, deg.	C…Cg, Å
C13-H13A…Cg (1-*x*, -*y*, 1-*z*) (Cg is centroid of the pyrazol ring N3N4C19C20C18), intramolecular	2.94	2.89	10.86	135	3.692(19)

Table S3. Selected parameters of π-π intermolecular interactions in $H_3O[Ln(Q^{cy})_4]$ (Ln = Nd, Sm, Eu, Gd, Tb) and $[AgGd(Q^{cy})_4]_n$ (the analysis was done using PLATON software).

Aromatic fragment I	Aromatic fragment J [symmetry index]	C_g-C_g, Å[a]	α, deg. [a]	β, deg.[a]	γ, deg.[a]	C_g(I)_Perp, Å[a]	C_g(J)_Perp, Å[a]	Slippage
				**Eu**				
N1N2C2C3C1 (pyrazole)	N3N4C19C20C18 (pyrazole) [1-*x*,*y*,1/2-*z*]	3.678(4)	17.3(4)	19.8	20.7	3.440(3)	3.461(3)	1.243
				**Gd**				
N1N2C10C8C9 (pyrazole)	N3N4C27C25C26 (pyrazole) [1-*x*,*y*,1/2-*z*]	3.633(4)	16.7(4)	20.0	21.1	3.389(3)	3.415(3)	1.240
				**Tb**				
N1N2C3C2C1 (pyrazole)	N3N4C20C19C18 (pyrazole) [1-*x*,*y*,1/2-*z*]	3.622(2)	17.1(2)	19.9	21.2	3.3776(18)	3.4052(17)	1.235
				**Ag-Gd**				
N1N2C2C3C1 (pyrazole)	C38-C43 (phenyl) [1-*x*, 1-*y*, 1-*z*]	3.630(9)	6.0(8)	8.4	14.4	3.517(6)	3.591(7)	0.529
N5N6C36C37C35 (pyrazole)	C12-C17 (phenyl) [1-*x*, 1-*y*, 1-*z*]	3.737(9)	10.3(8)	14.9	23.4	3.432(5)	3.612(7)	0.960

[a] C_g-C_g = distance between ring centroids (Å); α = dihedral angle between planes I and J (deg.); β = angle C_g(I)->C_g(J) or C_g(I)->Me vector and normal to plane I (deg.); γ = angle C_g(I)->C_g(J) vector and normal to plane J (deg.); C_g(I)_Perp = Perpendicular distance of Cg(I) on ring J (Å); C_g(J)_Perp = Perpendicular distance of Cg(J) on ring I (Å).

Table S4. Continuous Shape Measures (CShM) values [98] for the potential coordination polyhedron of Ln in $H_3O[Ln(Q^{cy})_4]$ (Ln = Eu, Gd, Tb) and $[AgGd(Q^{cy})_4]_n$

Complex/ Polyhedron	**Eu**	**Gd**	**Tb**	**Ag-Gd**
Octagon, D_{8h}	29.077	28.977	28.662	29.070
Heptagonal pyramid, C_{7v}	23.862	23.910	24.016	22.182
Hexagonal bipyramid, D_{6h}	17.257	17.350	17.353	16.782
Cube, O_h	9.836	9.872	10.073	9.624
Square antiprism, D_{4d}	0.322	0.254	0.246	0.322
Triangular dodecahedron, D_{2d}	1.563	1.691	1.701	2.471
Johnson gyrobifastigium, D_{2d}	15.306	15.648	15.367	16.928
Johnson elongated triangular bipyramid, D_{3h}	29.006	29.140	28.938	27.177
Biaugmented trigonal prism, C_{2v}	2.347	2.389	2.312	2.654
Biaugmented trigonal prism J50, C_{2v}	1.916	1.946	1.904	2.357
Snub diphenoid, D_{2d}	3.907	4.080	3.950	5.045
Triakis tetrahedron, T_d	10.438	10.506	10.677	10.294
Elongated trigonal bipyramid, D_{3h}	24.018	24.259	24.290	23.361

Table S5. Electronic transitions for all the complexes.

$H_3O[Sm(Q^{cy})_4]$, $[AgSm(Q^{cy})_4]_n$	Range, nm	Energy, cm^{-1}
$^4G_{5/2} \rightarrow {}^6H_{5/2}$	550-570	17880
$^4G_{5/2} \rightarrow {}^6H_{7/2}$	570-620	16820
$^4G_{5/2} \rightarrow {}^6H_{9/2}$	630-670	15160
$^4G_{5/2} \rightarrow {}^6H_{11/2}$	680-730	14270
$^4G_{5/2} \rightarrow {}^6H_{13/2}$	780-810	12860
$^4G_{5/2} \rightarrow {}^6F_{1/2}$	880-895	11500
$^4G_{5/2} \rightarrow {}^6H_{15/2}$	895-910	11400
$^4G_{5/2} \rightarrow {}^6F_{3/2}$	920-940	11350
$^4G_{5/2} \rightarrow {}^6F_{5/2}$	940-970	10770
$^4G_{5/2} \rightarrow {}^6F_{7/2}$	970-1050	9930
$^4G_{5/2} \rightarrow {}^6F_{9/2}$	1100-1200	8870
$H_3O[Tb(Q^{cy})_4]$, $[AgTb(Q^{cy})_4]_n$	Range, nm	Energy, cm^{-1}
$^5D_4 \rightarrow {}^7F_6$	475-500	20440
$^5D_4 \rightarrow {}^7F_5$	545-555	18390
$^5D_4 \rightarrow {}^7F_4$	575-600	17410
$^5D_4 \rightarrow {}^7F_3$	610-630	16140
$^5D_4 \rightarrow {}^7F_2$	630-660	15460
$^5D_4 \rightarrow {}^7F_1$	670-675	15010
$^5D_4 \rightarrow {}^7F_0$	675-680	14890
$H_3O[Dy(Q^{cy})_4]$, $[AgDy(Q^{cy})_4]_n$	Range, nm	Energy, cm^{-1}
$^4F_{9/2} \rightarrow {}^6H_{15/2}$	450-500	22250
$^4F_{9/2} \rightarrow {}^6H_{13/2}$	555-600	18790
$^4F_{9/2} \rightarrow {}^6H_{11/2}$	645-675	16460
$^4F_{9/2} \rightarrow {}^6H_{9/2}$	740-770	14600

$^4F_{9/2} \rightarrow {}^6H_{5/2}$	930-1030	12120
$^4F_{9/2} \rightarrow {}^6F_{5/2}$	1120-1205	9860
$^4F_{9/2} \rightarrow {}^6F_{3/2}$	1265-1310	9080
$^6H_{9/2} + {}^4F_{11/2} \rightarrow {}^6H_{15/2}$	1370-1405	~7550
$H_3O[Eu(Q^{cy})_4]$, $[AgEu(Q^{cy})_4]_n$	Range, nm	Energy, cm^{-1}
$^5D_0 \rightarrow {}^7F_0$	560-565	17290
$^5D_0 \rightarrow {}^7F_1$	575-600	16920
$^5D_0 \rightarrow {}^7F_2$	610-630	16270
$^5D_0 \rightarrow {}^7F_3$	645-665	15430
$^5D_0 \rightarrow {}^7F_4$	690-705	14470

Table S6. Color coordinates for all the complexes

Complex	X	Y
$H_3O[Sm(Q^{cy})_4]$,	0.61	0.35
$[AgSm(Q^{cy})_4]_n$	0.61	0.39
$H_3O[Tb(Q^{cy})_4]$,	0.33	0.60
$[AgTb(Q^{cy})_4]_n$	0.34	0.61
$H_3O[Dy(Q^{cy})_4]$,	0.41	0.44
$[AgDy(Q^{cy})_4]_n$	0.43	0.47
$H_3O[Eu(Q^{cy})_4]$,	0.66	0.34
$[AgEu(Q^{cy})_4]_n$	0.66	0.34

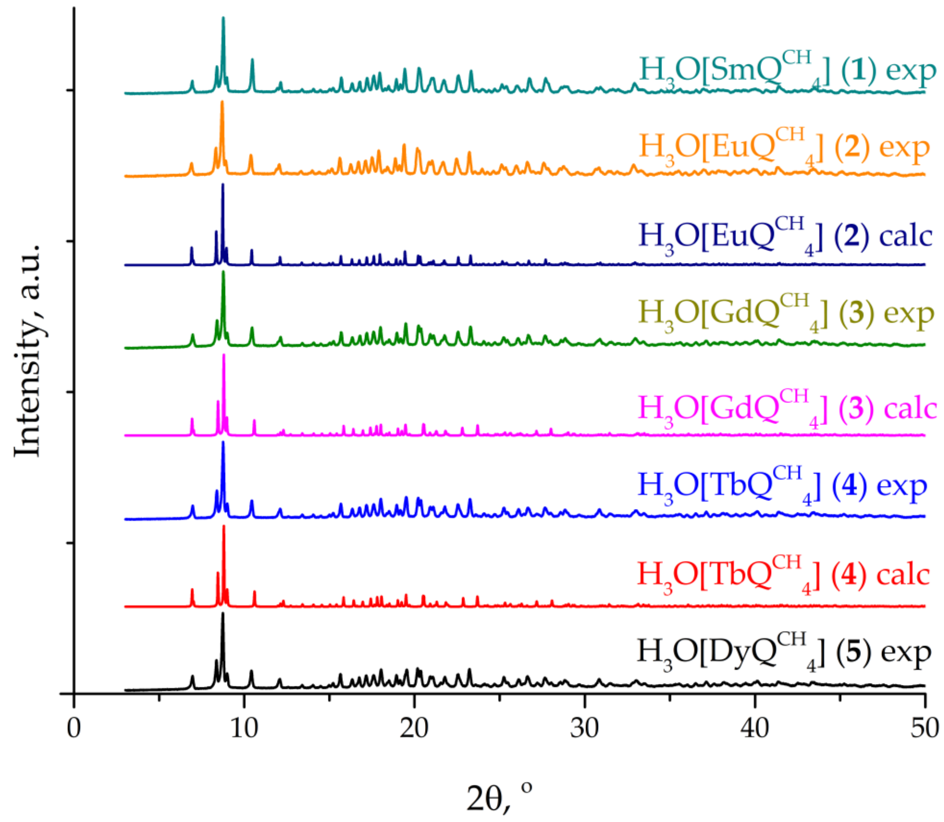

Figure S1. PXRD patterns of 1-5 and simulated from single crystal data of 2, 3 and 4.

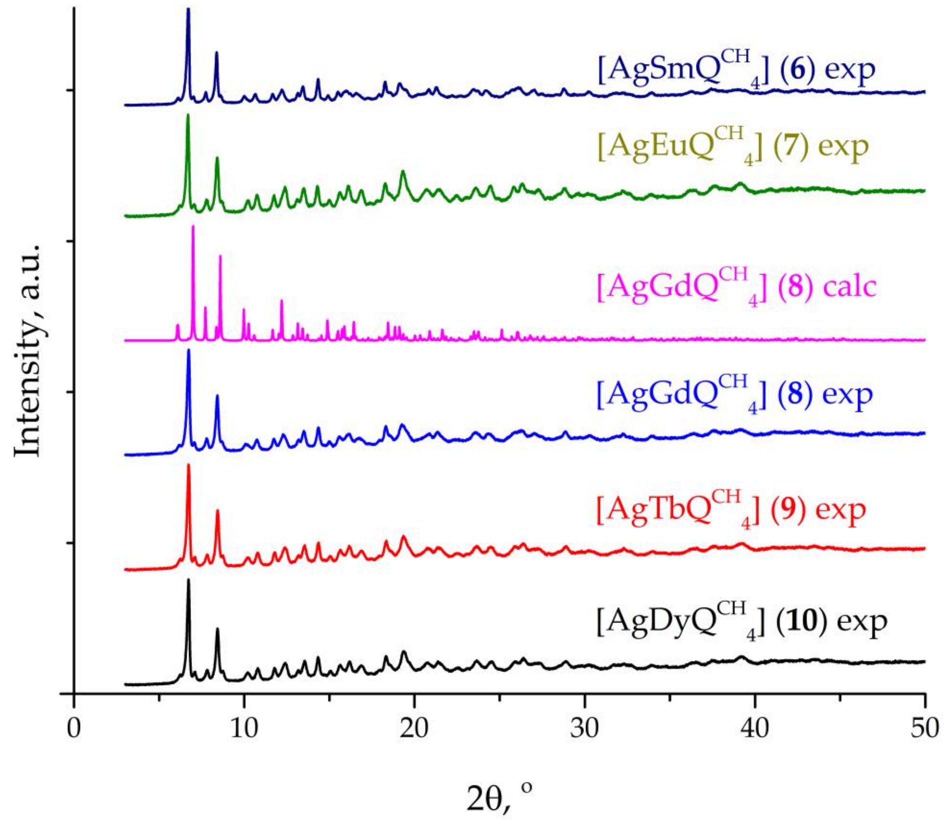

Figure S2. PXRD patterns of 6-10 and simulated from single crystal data of 8.

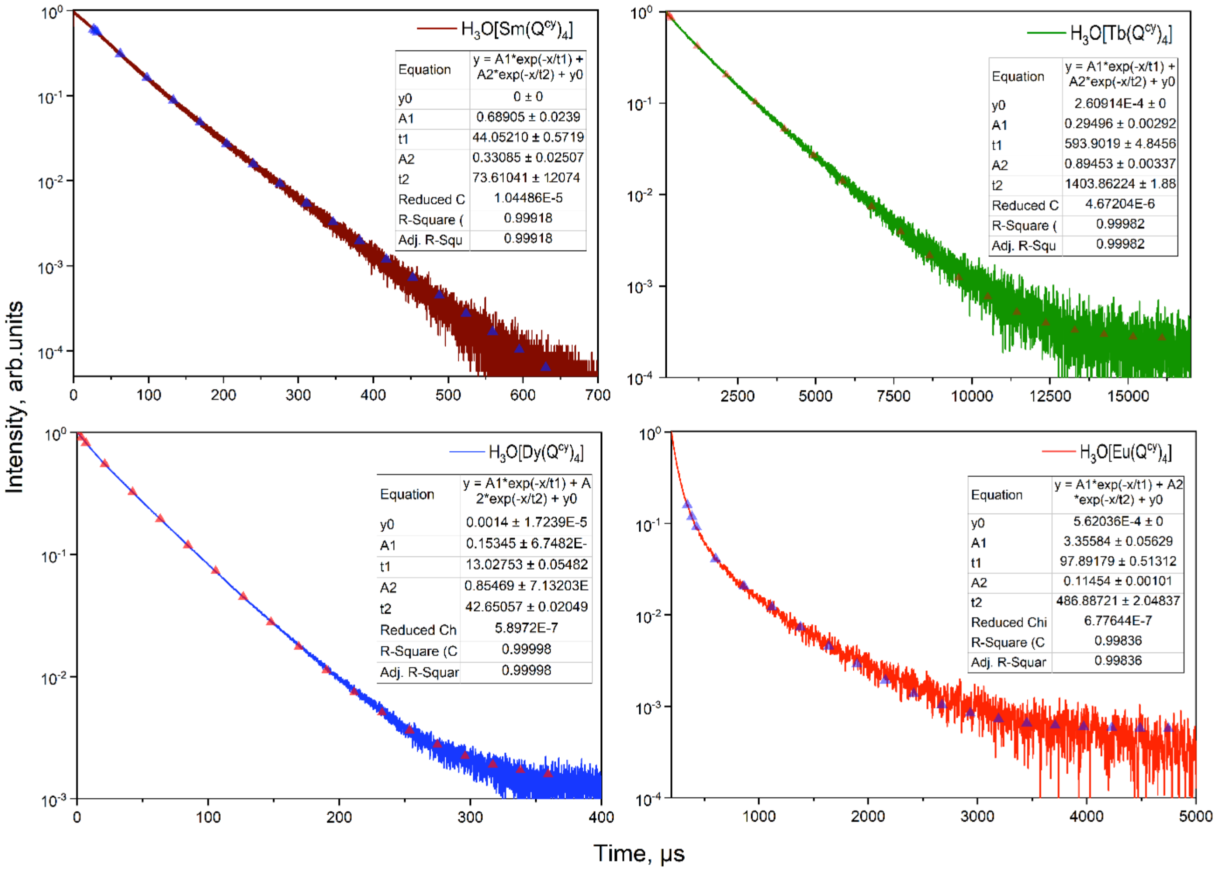

Figure S3. PL decays for the complexes containing H_3O^+

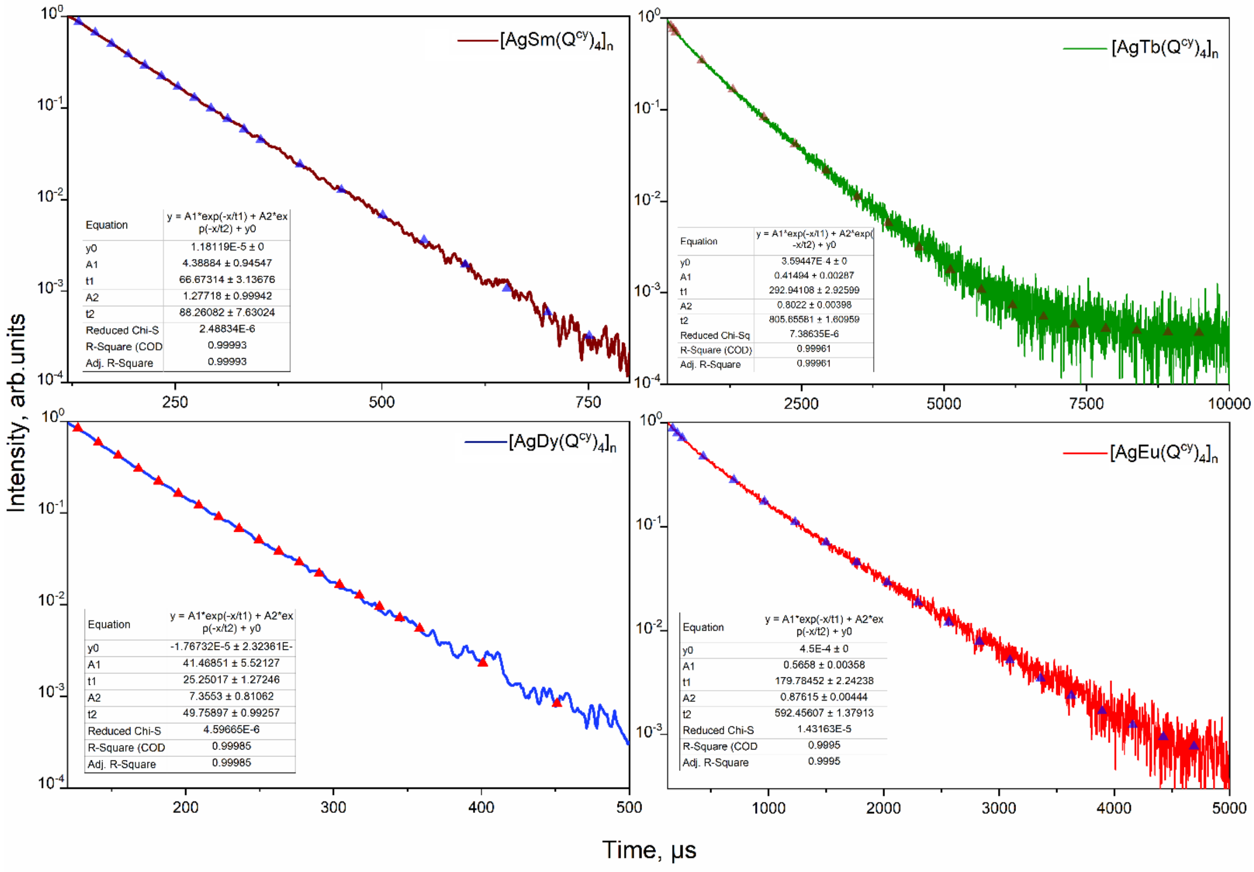

Figure S4. PL decays for the complexes containing Ag^{+}

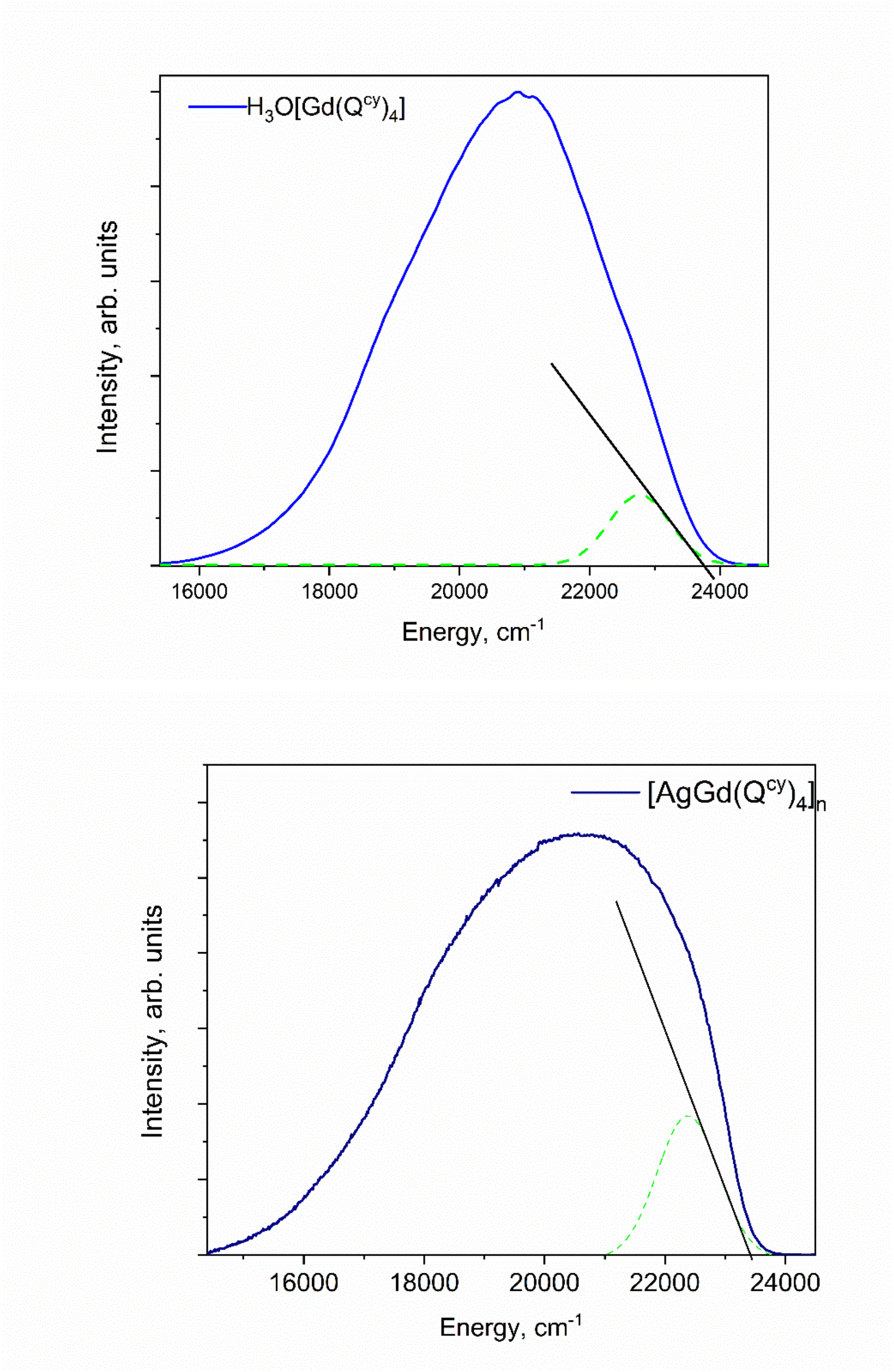

Figure S5. Phosphorescence spectrum of Gd^{3+} complexes at 77K

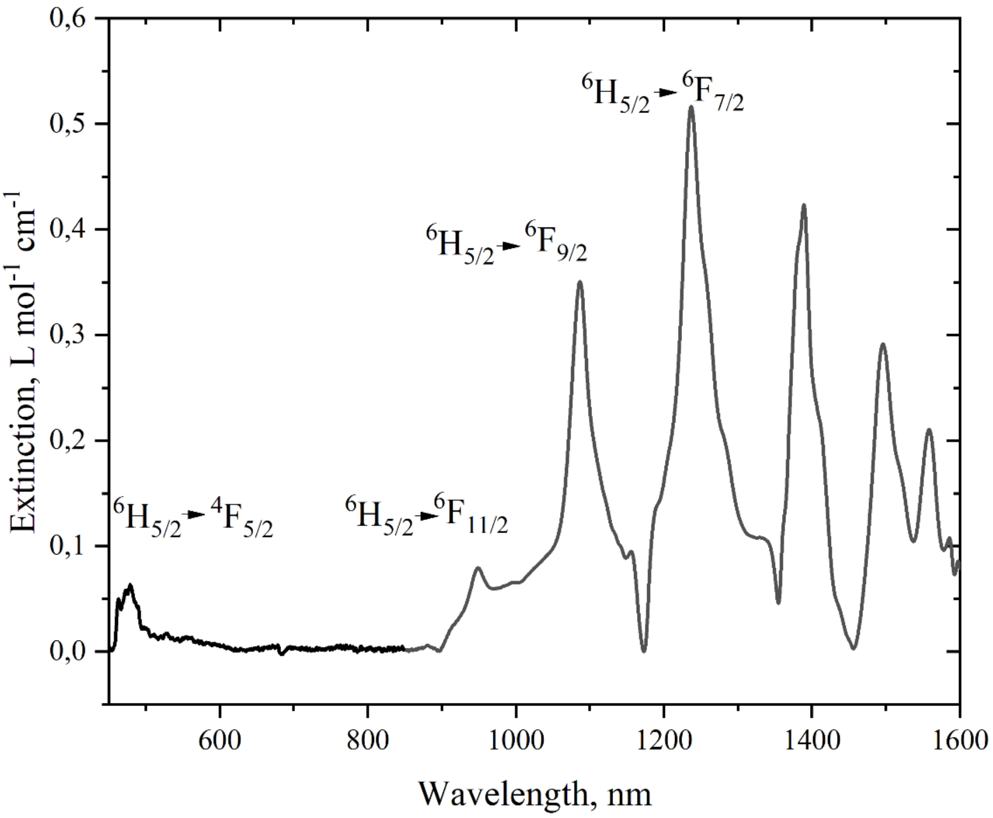

Figure S6: Absorption spectra for DMSO solution of complex $H_3O[Sm(Q^{cy})_4]$ (1) with concentration $3{\cdot}10^{-3}$ M
